# The Amending Bill

**Published:** 1913-07-19

**Authors:** 


### OUR NATIONAL INSURANCE ACT DEPARTMENT.

### LXI.—The Amending Bill (*Concluded*).

### Casual Employment.

It was a foregone conclusion that the Government would make proposals in the first Amending Bill to modify the provisions of the Act in regard to persons in casual employment. This has indeed proved to be the case, but it cannot be said that any satisfactory solution of the difficulty has yet been attained. Power is to be conferred on the Insurance Commissioners to enable them to modify the provisions of the Act in its application to persons whose employment is of a casual or intermittent nature. The only indication as to how this is to be done is conveyed in the instruction to the Insurance Commissioners that they may, if they please, adapt the provisions of the Act so that the employer's contributions shall not exceed 6d. in any week, nor the employed contributor's 4d. (in the case of a woman 3d.).

Thus the ultimate solution of this difficult question is left to the ingenuity and discretion of the Insurance Commissioners.

### Offences Against the Act.

There is one other proposal in the Amending Bill which is of material interest to insured persons. This proposal is contained in Clause 11, by which it is to be declared an offence against the Act if an employer deducts or attempts to deduct from the wages of an employed contributor the whole or any part of the employer's contribution. This is designed to cover the case of persons who are in receipt of low wages and whose own contribution should therefore be less than the normal 4d. or 3d., as the case may be. The provision is a useful addition to the powers of the Insurance Commissioners, but there is little doubt that if a case had been tried it would have been found that the existing powers of the Commissioners were sufficient to effect the same object.

At present if contributions are paid on the lower scale applicable to those insured persons over twenty-one years of age, who are in receipt of low wages within the prescribed limits, the contribution cards must be accompanied by "low-wage declarations." when they are sent in at the end of the quarter to the approved societies. There is really no difficulty in obtaining these forms or in filling them up when procured, as they have been made as simple as the necessities of the case will permit.

To ill-educated persons and to those whose knowledge of Acts of Parliament is but rudimentary the necessity for applying for special forms and for filling them up is not readily understood. It is hardly to be wondered at in the circumstances that such persons have not appreciated the relief accorded to them by the complicated provisions of the National Insurance Act, even in the simplified way in which they are expressed on the Insurance cards and books.

Employers are apparently entitled under present regulations of the Insurance Commissioners to deduct the contribution at the full rate of 4d. (or 3d. in the case of women) from the weekly wage, and to leave the employee to appeal to the Commissioners where he feels aggrieved and thinks that relief could be claimed. The result has been—in agricultural districts especially—that the insured persons have been called upon to pay a higher contribution than that required from them under the provisions of the Act. It is this state of affairs which the proposed amendment is designed to rectify. By sub-section 2 of the same clause a time limit of one year from the date of offence is proposed during which it will be possible for legal proceedings to be instituted. If an offence be proved, the employer will have to make good to the Insurance Commissioners a sum equal to the total amount of all the contributions which he has failed or neglected to pay.

### Decision of Disputes.

Closely akin to the provisions relating to offences against the Act is the question of the settlement of disputes between interested persons or parties. The Act itself provides machinery for the decision of certain types of dispute between insured persons and approved societies and Insurance Committees respectively. This machinery is in the nature of arbitration, with right of appeal to the Insurance Commissioners. The occasions on which it has been necessary to put the machinery into motion have up till now been remarkably few, so far as can be gathered, but disputes of other kinds have, unfortunately, been more frequent and, curiously enough, there was no provision made in the Act for their settlement.

Disputes have arisen between approved societies in regard to claiming members and to the transfer of members from one society to another. Disputes have also occurred between approved societies and Insurance Committees in regard to the liability of the latter for the administration of medical and sanatorium benefits. Satisfactory settlements have been made in most cases by the action of the Insurance Commissioners, but they really had no power to enforce their views. It is therefore proposed in the Amending Bill (Clause 8) to institute the procedure, already provided under the Act for disputes between members and their societies, for the settlement of the larger class of disputes which arise, and must continue to arise, between approved societies and Insurance Committees among themselves.

This clause is therefore designed to rectify an important omission from the original structure of the Act which had made itself apparent as the result of experience.

### Powers of the Joint Committee of the Commissioners.

Another omission of a similar kind has made itself manifest to those with high legal training, for it now appears that the Joint Committee of the Insurance Commissioners has no power under the Act to issue regulations or to affix a seal thereto. The omission, which is, of course, of a technical character, was the result of the adoption of the amendments relating to the separation of the four Commissions without fully realising the full extent of such a fundamental alteration.

In spite of this lack of authority the Joint Committee have actually promulgated several orders, and Clause 10 of the Amending Bill is so drawn as to render such previously issued orders valid as well as to confer the power of issuing valid orders in future.

### Extension of the Powers of the Insurance Commissioners.

The remaining operative proposals of the Amending Bill are designed to extend the powers of the Insurance Commissioners. They are to be allowed under Clause 12 to revoke, vary, or amend any order that they have previously made—a very necessary provision, seeing that even the Commissioners are not infallible. Under Clause 9 they are to be given power to make regulations regarding the several matters that are set out in a list in the schedule to the Bill.

Several of these matters will become important in the not distant future, for among other things the Commissioners will be empowered to make regulations as to the conditions upon which approved societies or branches of such societies may be amalgamated and as to the financial adjustments necessitated thereby.

The extraordinarily large number of societies and branches of societies that have obtained approval cannot all be expected to maintain a separate existence indefinitely, and it is clear that many will become absorbed in the friendly orders, or will be taken over by one or other of the large approved societies formed by the collecting friendly societies and industrial life assurance companies.

Voluntary dissolution is also provided for, and the terms upon which the contracts may be taken over by other societies will be regulated by orders of the Commissioners, to whom power is also to be given to determine the basis of valuation of the liabilities and assets.

Compulsory dissolution by the withdrawal of approval is also adumbrated. It may be remarked in passing that some few societies already appear to be in such a condition as to require the immediate exercise of this power of cancellation by the Commissioners.

The two clauses (Clauses 9 and 12) which refer to these extended powers of the Insurance Commissioners are non-controversial, and will doubtless pass all stages of Parliamentary procedure without material alteration, unless, indeed, it be objected that the matters should be regulated directly by Act of Parliament.

### Further Suggested Amendments.

It must not be supposed that all the clauses will share the same freedom from alteration, even though on the whole, as we have already said, the Bill is generally acceptable.

The chief criticism of the Bill is on account of its omissions. At the inaugural meeting of the National Conference of Industrial Assurance Approved Societies held on July 4, it was apparently the unanimous opinion of all present that the time has not yet arrived for amendments other than those foreshadowed in the Bill. This opinion is not held by the officials of approved societies formed by the friendly orders. Mr. A. H. Warren, President of the National Conference of Friendly Societies, has called attention in a very trenchant letter published in the *Times* on July 11 to the effect of the National Insurance Act on the finances of the friendly societies. He claims that the experience of the past six months has falsified the financial basis of the Act, because the actuarial estimates were made on the assumption that the sickness rates shown in the past by the friendly societies would be reproduced under the National Insurance Scheme in the future, whereas as a matter of fact he can show instances where the claims for benefit this year have been 50 and even 70 per cent. in advance of the corresponding months last year.

Such a claim, and from so high an authority, is bound to receive attention, but whether it will be possible to incorporate provisions in the present Bill to protect the societies from the absolute ruin which he predicts unless drastic steps are taken is a matter on which we have grave doubt.

We should like to see a clause inserted which would make the maternity benefit the property of the wife, and in order to enable the benefit to be administered promptly the section of the Act which makes the fees of a doctor who is called in by a midwife a charge on the maternity benefit should, in our opinion, be repealed.

